# Exercising for Insulin Sensitivity – Is There a Mechanistic Relationship With Quantitative Changes in Skeletal Muscle Mass?

**DOI:** 10.3389/fphys.2021.656909

**Published:** 2021-05-12

**Authors:** Jasmine Paquin, Jean-Christophe Lagacé, Martin Brochu, Isabelle J. Dionne

**Affiliations:** ^1^Research Centre on Aging, Affiliated With CIUSSS de l’Estrie-CHUS, Sherbrooke, QC, Canada; ^2^Faculty of Physical Activity Sciences, University of Sherbrooke, Sherbrooke, QC, Canada

**Keywords:** insulin sensitivity, muscle mass, muscle quality, resistance training, muscle hypertrophy, muscle capillarization, muscle mitochondrial activity, muscle mitochondrial biogenesis

## Abstract

Skeletal muscle (SM) tissue has been repetitively shown to play a major role in whole-body glucose homeostasis and overall metabolic health. Hence, SM hypertrophy through resistance training (RT) has been suggested to be favorable to glucose homeostasis in different populations, from young healthy to type 2 diabetic (T2D) individuals. While RT has been shown to contribute to improved metabolic health, including insulin sensitivity surrogates, in multiple studies, a universal understanding of a mechanistic explanation is currently lacking. Furthermore, exercised-improved glucose homeostasis and quantitative changes of SM mass have been hypothesized to be concurrent but not necessarily causally associated. With a straightforward focus on exercise interventions, this narrative review aims to highlight the current level of evidence of the impact of SM hypertrophy on glucose homeostasis, as well various mechanisms that are likely to explain those effects. These mechanistic insights could provide a strengthened rationale for future research assessing alternative RT strategies to the current classical modalities, such as low-load, high repetition RT or high-volume circuit-style RT, in metabolically impaired populations.

## Introduction

Skeletal muscle (SM) is the most important component of fat-free mass (FFM) and plays a key role in overall metabolic health. SM accounts for up to 80% of whole-body glucose consumption under insulin-stimulated conditions (DeFronzo and Tripathy, 2009; Honka et al., 2018) and is thought to contribute to as much as 25% of basal metabolic rate (Dulloo et al., 2012). Furthermore, the existing body of research on components of total daily energy expenditure supports a robust correlation between total FFM and resting energy expenditure (Tam et al., 1999; Illner et al., 2000; Blanc et al., 2004). The onset of insulin resistance (IR) at the SM level have been suggested to initiate the development of IR, which if not managed could lead consecutively to glucose intolerance and then type 2 diabetes (T2D) (Petersen and Shulman, 2002; Roden and Shulman, 2019), a multifactorial metabolic and endocrine disease with high individual and public health consequences (Rosenquist and Fox, 2009). Supporting the role of SM in cardiometabolic health, multiple observational have repetitively demonstrated an association between low relative FFM and cardiometabolic complications (Srikanthan and Karlamangla, 2011; Lee et al., 2018; Zhang et al., 2018). In older individuals, sarcopenia as defined by musculoskeletal decline (Cooper et al., 2012) has shown itself to be associated with altered glucose homeostasis (Yang et al., 2017; Shou et al., 2020) as well as autonomy and overall quality of life (Tsekoura et al., 2017).

Exercise, by inherently enhancing SM metabolism and function, has proven itself to be incredibly efficient for whole-body glucose homeostasis management and overall cardiometabolic health (Umpierre et al., 2011; Bird and Hawley, 2017). Likewise, the exercise physiology community also agrees on the importance of maintaining an optimal degree of SM tissue mass for exercise capacity and overall autonomy (Yang, 2014). Results from seminal studies investigating the impact of resistance training (RT) and concurrent quantitative changes of SM mass on whole-body glucose homeostasis suggested an interplay between SM hypertrophy and SM glucose disposal rates (Yki Jarvinen and Koivisto, 1983; Miller et al., 1984; Eriksson et al., 1997, 1998). These studies, which have been frequently replicated since, have contributed to the hypothesis that RT targeting SM hypertrophy could be as efficient as aerobic exercise performed alone for improving SM insulin sensitivity (IS) (Dela and Kjaer, 2006; Pesta et al., 2017; DiMenna and Arad, 2018). Additionally, according to the role of SM glycogen synthesis in IS through Akt signaling (Russell, 2010; Kleinert et al., 2013; Yang, 2014), it could be inferred that increasing total SM mass will lead to a greater storage capacity (Shepherd et al., 2014). Collectively, it has been suggested that exercising toward SM hypertrophy (or maintenance of it in a context of weight loss) can therefore protect against metabolic syndrome and other metabolic diseases related to energy surplus (Ravussin and Bogardus, 1990; Wood et al., 2012).

However, difficult they are to identify, a certain number of studies have postulated a divergence between SM hypertrophy *per se*, and intrinsic changes in SM IS or have pointed out a lack of clarity around the mechanisms involved (Holten et al., 2004; Bird and Hawley, 2017; Pesta et al., 2017). Furthermore, the relationship between relative or absolute FFM and glucose homeostasis is inconsistent across studies (Gippini et al., 2002; Goulet et al., 2007; Glouzon et al., 2015; Perreault et al., 2016; Stuart et al., 2017). Amongst these studies, Brochu et al. (2008) have shown in non-diabetic postmenopausal women matched for fat mass that those displaying a larger FFM had higher fasting insulin and glucose compared to those with lower FFM (Brochu et al., 2008). Furthermore, the results of Glouzon et al. (2015) show that following a 6-month mixed-exercise intervention, overweight to obese women who had lost FFM had better improvement in HOMA-IR compared to women who had gained muscle mass, independently of visceral adipose tissue and fat mass percentage (Glouzon et al., 2015). Although it is impossible to infer a causal relationship, or lack thereof, between SM hypertrophy and glucose homeostasis improvements, these results are favorable to the idea that exercise-induced muscle metabolic changes in favor of IS and SM hypertrophy might be independent. A recent systematic literature review addressing the comprehensive effects of physical activity on IS conducted by Bird and Hawley (2017) stated the mechanisms that underpin the effects of SM mass quantitative changes on IS are not fully understood, given the disparity in the current body of research on RT and glucose homeostasis in humans (Bird and Hawley, 2017).

Given the plausible independence between SM hypertrophy and whole-body glucose homeostasis improvements, a closer look at the disparities in different populations and at the mechanistic insights behind these discrepancies is warranted. The current review will attempt to identify some of the mechanisms that could be involved with a specific and narrow focus on exercise intervention studies. Hence, the first section will highlight the present level of evidence supporting SM hypertrophy as a way to enhance whole-body glucose homeostasis as. The second and third sections will review some of the plausible mechanistic explanations of the relationship between quantitative changes of SM mass and IS.

## Narrative Literature Review

### Glucose Homeostasis, Exercise, and Skeletal Muscle Hypertrophy: Previous and Current State-of-the-Art

Miller et al. (1984) pioneered the research domain of exercise-induced SM hypertrophy and concurrent improvement in IS by demonstrating a significant association between RT-induced SM mass increases and a lower insulin area under the curve during a glucose tolerance test in 10 inactive, but otherwise healthy, male college students following a 10-week intervention. A decade later, the same group (Miller et al., 1994) reported similar results in a sample of 11 men in their fifties and early sixties. The 16-week intervention led to an improvement in IS, measured with the gold standard euglycemic-hyperinsulinaemic clamp, together with a concomitant 1.2 kg average SM increase. Another seminal study that has shaped the field is the one by Eriksson et al. (1997) who reported conclusions comparable to those of Miller et al. Their results support a strong and negative association (*r* = –0.73; *p* < 0.05) between increased SM mass and HbA1c in middle-aged men and women diagnosed with T2D in response to 3 months of circuit-style RT (Eriksson et al., 1997). Since, a growing body of evidence support circuit-style RT as an effective training modality to enhance metabolic health components, such as whole-body glucose homeostasis (Kolahdouzi et al., 2019) body composition and physical fitness (Kim et al., 2018) and pro-inflammatory cytokine profile (Kolahdouzi et al., 2019). One clear advantage of such a training protocol is its similarity to aerobic exercise from the perspective of the broad continuum of the energy systems, according to its ability to induce an elevated cardiovascular response (Gotshalk et al., 2004).

Given the irrefutable relevance of increasing peripheral IS in prediabetic and T2D populations, a large number of studies have conducted exercise trials in those populations in an attempt to identify the optimal RT exercise prescription (Praet and Van Loon, 2009). Cuff et al. (2003), compared the impact of 16 weeks of either AT alone, RT alone or a combination of the two on hyperinsulinemic-euglycemic clamp-derived IS and CT-scan derived SM cross-sectional area in a sample of 28 postmenopausal women with T2D (Cuff et al., 2003). Changes in SM cross-sectional area, as well as SM normal density (therefore fat-free tissue) were both strongly correlated with changes in glucose uptake during the IS assessment. Cauza et al. (2009) robustly investigated the impact of a 4-month RT intervention in untrained men and women who fulfilled diagnosis criteria for T2D (Cauza et al., 2009). CT-scan derived muscle cross-sectional area showed significant increases in quadriceps size after the intervention. Furthermore, there were significant improvements in HbA1C and fasting blood glucose, lowered body fat percentage higher lower-limb strength. Unfortunately, none of these outcomes were significantly correlated with increases in quadriceps cross-sectional area. Findings in line with those of Cauza et al. (2009) have been replicated more than once and are not atypical. Mavros et al. (2013) compared two RT protocols – low intensity without load increase and high intensity with progressive load increase – in a cohort of older adults with T2D (Mavros et al., 2013). Their results revealed that, in the high-intensity group, changes in SM mass were significantly and inversely correlated with HOMA2-IR. Conversely, in the low intensity group, there was no such association. In addition, in the high-intensity group, only participants who had an increase in SM mass had a HbA1c reduction. Surprisingly, participants in the low intensity group whom SM mass increased did not show improvements in any glycemic control outcomes. In T2D individuals, SM quantitative changes in response to RT do not always occur and are presumably unpredictable despite the application of a proper training stimulus according to population studies. A systematic review conducted by Gordon et al. (2009) looking at RT as a metabolic health enhancing tool for individuals with T2D has concluded that the minority of studies analyzed (2 amidst the 24 listed papers) reported significant increases in SM as well as inconsistent improvements in SM intrinsic insulin signaling between trials (Gordon et al., 2009). Nonetheless, the aforementioned review strongly supports a favorable effect of RT on whole-body glycemic control, IS and SM strength in people with T2D, again displaying a discrepancy between SM improvements in IS and whole-body glucose homeostasis in response to RT. In sum, one plausible explanation of a higher efficiency of one exercise protocol over another might be total training load, i.e., the combined effect of intensity and volume, irrespective of the effect on SM quantity.

The association between increases in SM and glucose homeostasis may also be highly relative to inter-population variability. Table 1 provides a brief overview of highly cited studies investigating FFM quantitative changes and glucose homeostasis in response to either resistance or mixed training intervention, or a comparison of the two, in different population studies. Aside from a few exceptions (Cuff et al., 2003; Bucci et al., 2016), studies that report concurrent improvements in SM mass and IS or its surrogates are often conducted in either young male adults (Roberts et al., 2013; Cocks et al., 2014; Shepherd et al., 2014) or adolescents (Shaibi et al., 2006; Van Der Heijden et al., 2010; Lee et al., 2012). Amongst others, areas where counter-intuitive results have been found include post-menopausal women and women with PCOS. For instance, Kogure et al. (2016), using a 16-week RT intervention, showed a positive association between HOMA-IR and SM mass in middle-aged women with and without polycystic ovary syndrome (PCOS), both at baseline and after the intervention, suggesting worsening glucose homeostasis with increased SM mass (Kogure et al., 2016). Furthermore, the results of Glouzon et al. (2015) show that following a 6-month mixed-exercise intervention, overweight to obese women who had lost FFM had better improvement in HOMA-IR compared to women who had gained muscle mass, independently of visceral adipose tissue and fat mass percentage (Glouzon et al., 2015). Even more, the authors found a positive correlation between exercise-induced changes in SM mass index [lean body mass(kg)/height(m^2^)] and HOMA-IR after the intervention. Interestingly, the association was even stronger after controlling for visceral fat and relative fat mass (% of total body weight) changes. Indeed, women and men differ not only in physical attributes but also in their SM substrates metabolism (Ansdell et al., 2020). Accumulating evidence suggests a higher intrinsic IS in women’s SM, given a higher relative proportion of type I SM fibers with greater capillary density and fatty acid oxidation rates, as well as higher anti-inflammatory adipokine activity (mainly leptin and adiponectin) (Lundsgaard and Kiens, 2014). Indeed, these different metabolic features are thought to be in part driven by enhanced SM estrogen signaling (Ikeda et al., 2019) and adipose tissue partitioning (Mancuso and Bouchard, 2019). In contrast, Bucci et al. (2016) observed a significant effect of a 4-month RT program on MRI-derived SM mass and thigh muscle glucose uptake assessed with positron emission tomography (^18^F-FDG) in older frail women offspring of normal weight/lean or overweight/obese mothers (Bucci et al., 2016). Furthermore, there was a significant and positive correlation between increases in glucose uptake and increases in absolute SM mass. While indices of SM quality, such as absolute and relative strength, were not measured in the latter study, these results might suggest that increasing SM in the context of frailty, or pathological losses of SM such as sarcopenia may improve glucose homeostasis, as it has been suggested before (Lexell, 1995; Volpi et al., 2004; Phu et al., 2015).

**TABLE 1:** Summary of studies that has investigated fat-free mass quantitative changes and glucose homeostasis in response to either resistance or mixed training intervention or a comparison.

Study	Intervention (Modality)	Sample	FFM and FM changes	Glucose homeostasis	Association
Miller et al., 1984	10-week, 3/week (10 exercises, 3 sets of 8 REPS)	10 Young male college students	↑ FFM ↔ FM	↓ OGTT insulin AUC	Positive association: ↑ FFM and ↓ OGTT insulin No association: ↑ FFM and ↓ OGTT glucose
Miller et al., 1994	16-weeks, 3/week (14 exercises, 3 to 15 REPS)	11 Healthy older (58 ± 1 years) men (BMI = 26.9 ± 1.0)	↑ FFM ↓ FM	↔ F-Glucose and Glucose OGTT ↓ F-insulin and insulin AUC ↑ Glucose disposal during clamp	No association shown.
Bucci et al., 2016	4-months, three times/week	Older frail women offspring of normal weight/lean mothers (*n* = 20) and or overweight/obese mothers (*n* = 17) Avg age: 72.3 and 71.5 years; Avg BMI: 26.6 and 27.9.	↔ Overall BC ↑ Quadriceps mass and adductor longus mass.	↔ F-Glucose and F-insulin ↑Muscle GU ↑ Whole-body IS (OOM group only)	Positive association: ↑ GU per kg and ↑ FFM
Roberts et al., 2013	12 weeks, 3/week (Progressive overload – from 2 sets of 12–15 to 3 sets of 6–8 REPS)	28 Healthy young (avg 21.5 years) men, overweight/obese (avg BMI 30.9)	RT group: ↑ FFM ↓ Total FM ↓ Trunk FM	RT group: ↔ HbA1c ↑ GT (OGTT)	No association shown.
Shaibi et al., 2006	16-week, 2/week (Multiple sets, moderate intensity/high volume, multiple joint exercises)	11 overweight (Avg BMI 32.5) adolescent males (Avg 15.1 years)	RT group: ↑ FFM ↓ %BF Control group: ↑ FFM	RT group: ↑ IS (FSIGVTT) Control group: ↔	IS changes independent of BC changes.
Dionne et al., 2004	6 months, 3/week (9 exercises targeting major muscle groups, 3 sets of 10 REPS)	33 Young (27.8 ± 3.5 years) and 12 old (66.6 ± 4.9) women (avg BMI: young; 21.9 ± 2.3; old: 25.4 ± 2.6)	Young: ↑ FFM Old: ↑ FFM ↓ FM	Young: ↑ M (+38mg/min) Old: ↔ M	No significant improvement in young when reported relative to FFM.
Lee et al., 2012	3 months, 3/week AT (40–60 min, 50–70% VO_2_peak) or RT (10 whole-body exercises, 1–2 sets 8–12 REPS, 60% 1RM)	42 Obese adolescents (avg age: control = 14.8, AT = 15.2, RT = 14.6)	AT group: ↓ FM RT group: ↑ FFM ↓ FM Control group: ↔	AT group: ↔ GT (OGTT) ↔ IS RT group: ↔ GT (OGTT) ↔ ↑ IS Control group: ↔	No association shown.
Kogure et al., 2016	16 weeks, 3/week (Strength and hypertrophy focused)	45 Young (28 ± 5.4 years) [women with (*n* = 47) or without (*n* = 52)] PCOS	With PCOS: ↑ FFM Without PCOS: ↔	With PCOS: ↑ HOMA-IR Without PCOS: ↔	Positive association: FFM and HOMA-IR in PCOS and control at baseline and post-intervention. Changes in FFM (LM/height^2^) were independent of changes in HOMA-IR after the intervention.
Poehlman et al., 2000	6-months, 3 days/week AT (endurance-based) or RT (target intensity: 80% 1RM)	51 Premenopausal (age range 18–35 years; BMI < 26)	AT group: ↔ FFM and thigh CSA ↑ Muscle attenuation RT group: ↑ FFM and thigh CSA ↑ Muscle attenuation	AT group: ↑ M RT group: ↑ M	When IS was expressed relative to FFM (mg/kg FFM/min), it improved significantly only in the AT group.
Glouzon et al., 2015	6-month, 3/week (Mixed intervention)	48 Post-menopausal (avg age 60 ± 5.0 years) women	↑ FFM ↑ FFMI ↑ Appendicular FFM	↑ *F*-glucose ↔ *F*-insulin ↔ HOMA-IR	Baseline: Positive: ↑ FFMI and HOMA-IR Positive: ↑ appendicular FFMI and HOMA-IR Post-intervention: Positive: ΔFFMI and ΔHOMA-IR
Amankwaah et al., 2019	9-month, mixed intervention (RT 2 days/week, RT 1 day/week)	Middle-aged men (*n* = 69) and women (*n* = 83) with mild obesity (avg BMI 30.0 ± 2.7 kg/m^2^)	↑FFM ↓ FM	↑ *F*-glucose ↔ HOMA-IR	Increases in FFM did not predict changes in measured cardiometabolic health outcomes. Although the relation between IS and Δ FFM was significant when expressed relative to body weight, it was not when expressed absolutely or relative to baseline FFM.
Fukushima et al., 2016	6 months, 3 days/week (Mixed intervention)	92 Obese (BMI: 33.2 ± 4.6) women (avg age 40.9 ± 10.4 years)	↓ FFMI ↓ FM	↓ HOMA-IR ↓ *F*-glucose ↓ *F*-insulin	ΔHOMA-IR was inversely associated with Δ%FFM (relative to weight).
Cuff et al., 2003	16 weeks, RT or mixed intervention (AT: aerobic classes; RT: 5 exercises, 2 sets of 12 REPS)	28 postmenopausal women with T2D (avg age: control: 60 ± 2.9; AT+RT: 63.4 ± 2.2; AT: 59.4 ± 1.9)	Both groups: ↑ FFM (SM CSA) ↓ Low-density SM	AT: ↔ GIR Mixed: ↑ GIR	Positive association: ↑ SM CSA and ↑ GIR.

*AT, aerobic training; Avg, average; AUC, area under the curve; BMI, body mass index (kg/m^2^); BC, body composition; CSA, cross-sectional area; *F*-glucose, fasting glucose; F-insulin, fasting insulin; FFM, fat-free mass; FFMI, fat-free mass index (kg/m^2^); FM, fat mass; GIR, glucose infusion rate; GT, glucose tolerance; GU, glucose uptake; HOMA-IR, homeostatic model assessment for insulin resistance; M, hyperinsulinemic-euglycemic clamp-derived insulin sensitivity; IS, insulin sensitivity; OGTT, oral glucose tolerance test; REPS, repetitions; RM, maximal repetition; RT, resistance training; SM, skeletal muscle; T2D, type 2 diabetes.*

In weight loss trials, a great deal of attention is directed toward the importance of SM mass maintenance in order to counter any loss in resting metabolic rate and therefore, energy expenditure. It is hypothesized that the reduced energy expenditure due to reduced SM mass will contribute to weight regain and deterioration of body composition, a phenomenon coined fat overshooting. However, this fat overshooting phenomenon appears to be attenuated in obese individuals (Jacquet et al., 2020; Dulloo, 2021) and, accordingly, the risk of developing T2D (Zou et al., 2020) or cardiovascular diseases (Zou et al., 2019) does not appear to be greater with weight cycling in initially obese individuals. This discrepancy between normal weight and overweight to obese individuals could be attributable to the “less essential FFM” theory, which was suggested by Marks and Rippe (1996) as an explanation to the observed benefits of decreasing total FFM after weight loss in severely obese individuals (Marks and Rippe, 1996). The foundation of their theory was that a certain proportion of the high SM mass of obese individuals is “functionally inessential” due to a lessened relative quality. They also speculated that a high SM mass was associated with a lower density tissue in obese individuals, impaired strength-to-size ratio as well as a lower mitochondrial density and capillarization. Taken together, these factors compromised SM work capacity. Considering those speculations, although SM is a functional physiological reserve in many circumstances, more does not necessarily mean better. This may suggest a “ceiling effect” at a certain level of SM, wherein adding muscle mass does not provide further metabolic advantage. In line with this, a study from Ghachem et al. (2019) has identified a cut-off point of appendicular muscle mass index [7.02 muscle mass (kg)/height (m)^2^] in sedentary older women above which IS is significantly reduced(Ghachem et al., 2019). Fukushima et al. (2016) using a 6-month mixed-exercise and nutritional intervention in 92 middle-aged obese women found that fasting insulin and glucose decreased in those whose SM mass index (kg/m^2^) also decreased over the course of the intervention (Fukushima et al., 2016). Leon et al. (2013) also illustrated this point in 132 middle-aged women who participated in a 6-month diet and exercise program (Leon et al., 2013). Before the intervention, the authors noted that appendicular SM mass was proportional to the level of obesity and was also proportionally related to fasting insulin levels. Interestingly, changes in appendicular SM mass were moderately correlated (*r* = 0.337) to changes in fasting insulin after the intervention. In a large intervention study, Amankwaah et al. (2019) investigated the contribution of body composition changes to improvements in cardiometabolic health following a 9-month mixed exercise intervention (RT twice a week and AT once a week) in obese (BMI 30.0 ± 2.7 kg/m^2^) middle-aged men (*n* = 69) and women (*n* = 83) (Amankwaah et al., 2019). Unexpectedly, SM hypertrophy did not contribute to improvements in glucose homeostasis. Furthermore, the authors mentioned that the relationship between changes in SM mass and some cardiometabolic indices (HDL-Cholesterol and IS index) was inconsistent across different expressions of SM mass. On one hand, the relationship was significant when SM mass changes were expressed as a percentage of total body weight, while it was not the case when changes were expressed relatively to baseline (%) SM mass or in absolute values (kg). This suggests that the observed changes in cardiometabolic health indices were predicted by changes in fat mass rather than quantitative changes in SM.

Given the paucity of findings with regards to the implications of SM hypertrophy in response to exercise in different populations and study design, the exercise physiology community would likely benefit from a more in-depth understanding of the mechanisms involved in such adaptations, as well as their implications. Herein, we thus suggest moving the debate forward by examining if and how the presence or the absence of SM hypertrophy influences glucose delivery and utilization in response to exercise interventions. The reader is directed toward Table 2, which provides a brief overview of studies simultaneously reporting insulin-sensitizing SM metabolic properties, FFM quantitative changes and glucose homeostasis parameters changes in the context of exercise interventions.

**TABLE 2:** Summary of studies reporting insulin-sensitizing skeletal muscle metabolic properties, fat-free mass quantitative changes, and glucose homeostasis parameters.

Study	Intervention (Modality)	Sample	FFM and FM changes	Glucose homeostasis	SM metabolic properties	Association
Layne et al., 2011	8-week RT (progressive overload)	19 sedentary middle-aged individuals, with MetS (5F/10M) and control (5F/4M)	MetS: ↑ FFM ↔ FM Control: ↑ FFM (1.3kg) ↔ FM	MetS: ↔ Control: ↑ GIR (25%)	Mets: ↑ GLUT4 expression ↑ ATP synthase ↑ AMPK expression Control: ↑ ATP synthase ↑ GLUT4 expression ↑ PGC-1α ↑ AMPK expression	No association shown. Higher absolute FFM and type 2 fiber proportion in Mets participants pre- and post-intervention.
Andersen et al., 2003	3 months of de-training after a 3-month heavy RT	7 young (26 ± 1y) inactive men	↓ Quadriceps CSA after detraining	↔ F-glucose, insulin, c-peptide, [insulin] during clamp ↓ Whole-body M during the last 30min of the clamp (11 ± 4%)	↔ GLUT4 mRNA ↔ CS mRNA ↔ HAD mRNA ↔ GS mRNA ↔ Capillary density ↓ Glycogen content	No significant correlation between changes in leg glucose uptake rates and changes in muscle mas Type IIX fiber proportion increased in the detrained state.
Cocks et al., 2014; Shepherd et al., 2014 (shared sample)	6-week RT focused on hypertrophy and strength (9 exercises, 3 sets of 12 rep) IMTG breakdown during 1-h cycling ≈65% VO_2_peak	13 young (20 ± 1 years) lean (avg BMI: 24 ± 0.8) sedentary men	↑ FFM ↓ FM	↑ GH: ↑ Matsuda index ↓ OGTT glucose and insulin	↑ IMTG content and density in type I and type II fibers ↑ IMTG breakdown during 1-h cycling in type I and type II fibers ↑ COX expression ↑ SDH activity ↔ Capillary density, capillary contacts, eNOS^ser1177^ phosphorylation	No association discussed between FFM changes, SM metabolic properties changes and GH.
Iglay et al., 2007	12-weeks RT, 3/week (8 exercises, 2 sets of 8), coupled with either low or high protein diet	36 older (62.2 ± 2 years) men (*n* = 18) and women (*n* = 18)	High-protein group: ↑ FFM Low-protein group: ↑ FFM	Both groups: ↔HbA1c, ISI-composite, plasma glucose, insulin, C-peptide and HOMA-IR	Both groups: ↔ IRS-1, Akt ↑ Atypical protein kinase	Significant effect of RT on FFM and FM, independent of protein intake. BC changes were not correlated with changes in GH.
Holten et al., 2004	6-week, 3/week (One-leg RT)	10 men with T2D and 7 healthy controls	Both groups: ↑ FFM	Both groups: ↑ Leg glucose clearance	Both groups: ↑ GLUT4 content ↑ Insulin receptor ↑ GS synthase activity ↑ GS protein content ↑AKT (1/2) ↔ Oxidative enzymes (CS, LDH, HAD)	No association shown. Changes in muscle metabolic properties were likely to be independent of leg FFM quantitative changes.
Ferrara et al., 2006	10 to 12-week weight stabilization, followed by either AT or RT exercise for 6 months, 3 days/week.	21 Overweight or obese (avg BMI: 29.9 ± 0.7) middle aged and older men (50–79 years)	AT: ↑ FFM ↓ Thigh CSA ↓ Subcutaneous FM RT: ↑ FFM	AT: ↔ F-glucose ↔ F-insulin ↑ M (480 pmol/m^2^/min insulin infusion clamp) RT: ↔ F-glucose ↔ F-insulin ↑ M (480 pmol/m^2^/min insulin infusion clamp) ↑ Non-oxidative carbohydrate metabolism	AT: ↑ GS fractional activity ↔ CS, PI 3-k, glycogen content RT: ↑ GS fractional activity ↔ CS, PI 3-k, glycogen content	The effect of insulin on GS activity was significantly and 2.5-fold greater in the AT group.
Van Der Heijden et al., 2010	12 weeks RT, 2/week (2–3 sets of 8–12 repetitions, 3 sets of 15–20 during week 9–12)	12 obese (BMI: 35.3 ± 0.7) adolescents	↑ FFM ↑ Subcutaneous FM	↑ Hepatic IS; ↓ glucose production rate (SLIVGTT and glucose tracer infusion)	↔IMTG content	Neither FFM at baseline and post-exercise were correlated with peripheral and hepatic IS.

*Akt, protein kinase B (PKB); AMPK, AMP-activated protein kinase; AT, aerobic training; ATP, adenosine triphosphate; Avg, average; AUC, area under the curve; BMI, body mass index (kg/m^2^); BC, body composition; BF, body fat; C, citrate synthase; COX, cytochrome oxidase; CSA, cross-sectional area; eNOS^ser1177^, endothelial nitric oxide synthase at phosphorylation site 1177; F-glucose, fasting glucose; F-insulin, fasting insulin; FFM, fat-free mass; FFMI, fat-free mass index (kg/m^2^); FM, fat mass; GIR, glucose infusion rate; GLUT4, glucose transporter type 4; GS, glycogen synthase; GT, glucose tolerance; GU, glucose uptake; HOMA-IR, homeostatic model assessment for insulin resistance; HAD, 3-hydroxyacyl-CoA dehydrogenase; IMTG, intramuscular triglycerides; IRS-1, insulin receptor substrate-1; LDH, lactate dehydrogenase; M, hyperinsulinemic-euglycemic clamp-derived insulin sensitivity; OGTT, oral glucose tolerance test; PGC-1α, peroxisome proliferator-activated receptor gamma coactivator 1-alpha; PI 3-k, phosphatidylinositol 3-kinases; REPS, repetitions; RM, maximal repetition; RT, resistance training; SLIVGTT, stable-label iv glucose tolerance test; SM, skeletal muscle; T2D, Type 2 diabetes.*

### Skeletal Muscle Hypertrophy, Microvascularization and Glucose Homeostasis – Barrier or Enhancer?

Adequate perfusion is critical for efficient glucose delivery toward SM tissue. For instance, both the architecture of the microvasculature and its adaptability to vasodilation cues orchestrate glucose delivery from the circulation to the cytoplasm (Schalkwijk and Stehouwer, 2005; Sjøberg et al., 2017). Under those circumstances, the endothelium acts as the main “gate-keeper” for glucose delivery (Richey, 2013). The sophisticated network of mechanisms behind insulin-stimulated vasodilation and recruitment of the vasculature being beyond the scope of the current review, readers are directed toward other excellent reviews for a more extensive discussion of these topics (Cocks and Wagenmakers, 2016; Lenasi and Klonizakis, 2016; Olver and Laughlin, 2016).

Capillary rarefication applies a physical barrier to adequate substrate flow toward SM tissue and is consequently an early indicator of SM-IR (Lillioja et al., 1987). Conversely, exercise-induced increases in capillary density allows for enlargement of the diffusible surface area, which promotes greater IS at the SM level (Akerstrom et al., 2014; Prior et al., 2015). In response to chronic AT, SM capillarity indexes (i.e., capillary ultrastructure, capillary density, capillary-to-fiber ratio, etc.) and IS have been found to both increase in a positive and linear fashion (Prior et al., 2014; Cocks et al., 2016; Mortensen et al., 2019). On another hand, collective evidence on vascular adaptations to exercise suggests a plausible relationship between total SM mass, SM fiber-type characterization and capillary density indexes. Interestingly, the impact of SM hypertrophy *per se* on SM capillary architecture has only been explored by a few trials. In those studies, individuals with lower total SM mass had distinct characterizations of SM fiber type (Trappe et al., 1995). For example, capillary density has been found to be significantly higher in endurance athletes compared to age-matched powerlifting athletes with substantially higher SM mass (Tesch et al., 1984). In a previous study, Green et al. (1999) demonstrated a parallel increase in the number of capillaries in contact with each fiber type and SM fibers area after a 12-week high intensity RT regimen in young college students (Green et al., 1999). More recently, Holloway et al. (2018) investigated the temporal response of SM angiogenesis during RT-induced SM hypertrophy in a sample of 36 young men (Holloway et al., 2018). Not only did they see significant hypertrophy of type I and type II muscle fibers, but they also measured a concomitant increase in capillary-to-fiber ratio in type I muscle fibers. The findings from this study suggest SM hypertrophy may not be a limiting factor for SM angiogenesis in type I muscle fibers. Noteworthy, these conclusions were not supported by an association between the observed changes in fiber size and capillarization indexes. In the same line, interesting results from Snijders et al. (2017) demonstrated that baseline SM capillarity was a strong driver of SM hypertrophy and increases in satellite cell content after a RT intervention in healthy older men (Snijders et al., 2017). Contrasting with the results of Holloway et al. (2018), the intervention did not elicit any increase in capillary density, neither in type I nor type II muscle fibers, although SM hypertrophy was present. By the same token, a recent study of Moro et al. (2019) has shown that baseline SM capillarization was a strong driver of SM hypertrophy following RT in older adults (Moro et al., 2019). Overall, those findings suggest that improving SM perfusion capacity for glucose, insulin and other growth factors transport may be paramount to SM hypertrophy. The other side of the coin of these findings is that improving muscle quality first, by improving capillarity, could be the most efficient strategy regardless of the ultimate goal being SM hypertrophy, enhanced glucose homeostasis, or both.

Even though the aforementioned findings revealed important insights on how microvascular adaptations may influence SM mass quantitative changes, few investigations showed a comprehensive assessment of the implications for IS. Yet, only a few trials and reviews have previously shed light on potential hypotheses. A study by Cocks et al. (2014) examined the effects of a short-term RT intervention on SM capillary architecture indexes in a sample of eight sedentary young men (Cocks et al., 2014). They found no significant differences in either capillary contacts or number of capillaries per fiber after the intervention, although participants saw significant improvement in post absortive glucose tolerance. Previously, a review by Deschenes and Kraemer (2002) reported that RT-induced SM adaptation generally resulted in a reduction in relative capillarization when SM hypertrophy also occurs (Deschenes and Kraemer, 2002). However, according to a later review from Harris (2005) this interpretation is highly conditional to the ways in which muscle capillarity is expressed (Harris, 2005). When assessing capillarization as a matter of capillary contacts, hence the number of capillaries per myofiber, it rather seems that RT has a positive impact. It is generally accepted that these adaptations are the direct consequence of an elevated local oxygen (O_2_) demand from the SM – that is greater oxidative phosphorylation (OXPHOS) rates (Guyton et al., 2010, Chapter 7). These observations are in favor of the superiority of circuit-type RT above low-rep/high load RT for improving IS-related SM properties. Likely, the ideal RT prescription for improving microvascular function and SM-IS has not been identified yet (Olver and Laughlin, 2016).

In all likelihood, there exist an important interplay between SM angiogenesis and the mitochondrial biogenesis transcription factors. Irrefutably, PGC-1α transcription is likely one of the main drivers of mitochondrial biogenesis (Tiraby and Langin, 2005) and has been shown to play a significant role in microvascular reactivity and adaptations, through eNOS phosphorylation and vascular endothelial growth factor (VEGF) transcription pathways (Arany et al., 2008; Chinsomboon et al., 2009; Baldelli et al., 2014). Moreover, PGC-1α transcription has been shown to reflect Hypoxia Inducible Factor-1α (HIF-1α) activity, hence SM local O_2_ demand (Gorski and De Bock, 2019). Indeed, endurance exercise generates continuous blood flow elevation toward SM for adequate O_2_ and substrate delivery (Andersen and Saltin, 1985). As a response, the capillary network surface increases in order to enlarge the perfusion surface available. These adaptations seem highly dependent on PGC-1α transcription (Arany et al., 2008). As high-load, low-rep or hypertrophy-driven RT generally elicits short duration muscle contractions and an intermittent elevation of blood flow comparatively to AT, it may be an insufficient physiological stimulus to promote PGC-1α-driven angiogenesis. By the same token, results from Mortensen et al. (2019) suggested that improved vasodilator and insulin signaling mostly occur in SM undergoing a significant exercise volume (i.e., duration of stimuli application) (Mortensen et al., 2019). In their recent review, Olver and Laughlin (2016) provided in-depth analysis of the impact of different exercise modes on microvascular dysfunction in T2D (Olver and Laughlin, 2016) and stated that:

*[exercise] program that engages the most skeletal muscle and the most muscle fibers within each skeletal muscle (i.e., greatest increase in fiber recruitment from rest to exercise), given that the stimulus is applied for a sufficient duration of time, will generate the most widespread adaptations, leading to improvements in microvascular function and insulin sensitivity*.

Such a statement reinforces the hypothesis that RT solely centered on SM hypertrophy may not be the most appropriate stimulus for microvascular adaptations and SM IS. A unique study of Hansen et al. (2012) provided important insights on the respective impact of endurance-type RT and hypertrophy-focused RT regimens (Hansen et al., 2012). In short, they compared the two exercise modalities with regards to their effect on glucose metabolism in a sample of 18 older men and women with impaired glucose tolerance. Their results revealed that both exercise modalities had positive effects on baseline IS, but the endurance-type modality had an additional effect on pancreas’s beta-cell function.

As pointed out earlier, another important feature of efficient glucose delivery is the endothelium’s vasodilation capacity in response to either elevated insulin levels or a high metabolic demand induced by SM contraction (Cocks and Wagenmakers, 2016). In insulin resistant states, capillary blood volume and flow do not increase normally in response to insulin, which is likely to contribute to abnormal glucose homeostasis (Lenasi and Klonizakis, 2016). To date, the exact effect of SM hypertrophy on the SM microvasculature response to vasodilation physiological cues remains unclear, although RT has been found to exert significant benefits (Olver and Laughlin, 2016). Using an ultrasound method, Cohen et al. (2008) reported improvements in vasodilation of the forearm skin microvasculature in elderly T2D patients after a 14-month RT period (Cohen et al., 2008). Another recent study from Russell et al. (2017) using a 6-week RT protocol in T2D patients also showed significant improvements in muscle microvascular blood flow and a concomitant significant improvement in IS (Russell et al., 2017). Changes in fasting blood glucose, HbA1c and glucose area under the curve during an oral glucose tolerance test were all induced by a higher microvascular blood flow (adjusted for percent SM, body mass index, brachial blood flow and mean arterial pressure). Importantly, these changes were accompanied by a 44% increase in relative strength and an average of 1.3 kg SM gain. It is therefore legitimate to debate which one of those two improvements is more related to enhanced glucose homeostasis, relative strength being an important feature of muscle quality (Barbat-Artigas et al., 2013).

Additionally, the heterogeneity of blood flow distribution between muscle fiber types is likely to play an important role in the insulin-mediated vasoreactivity. Heinonen et al. (2015) reviewed this topic and reported a study by Behnke et al. (2011) that established that muscle fibers with lower oxidative potential, or known as type II fibers, are also prone to high adrenergic stimulation (Behnke et al., 2011; Heinonen et al., 2015). Thus, it is likely that these SM fibers display less upregulation of vasodilation mediated by endothelial factors, as well as an elevated α-adrenergic-mediated vasoconstriction, compared to type I fibers. Previous data suggested that obese (Krotkiewski et al., 1990), first-degree relatives (Nyholm et al., 1997), and T2D (Mårin et al., 1994) individuals might have a higher proportion of type II muscle fibers and second, usually hypertrophy-driven RT exercise has been hypothesized to mostly target type II muscle fibers (Folland and Williams, 2007; Netreba et al., 2013; Grgic and Schoenfeld, 2018). Admittedly, one could hypothesize that targeting SM hypertrophy might not be ideal in order to improve SM mechanisms of IS.

### New Insights in Mitochondrial Adaptations in Skeletal Muscle Hypertrophy

Amongst the vast array of insulin-sensitizing adaptations from chronic exercises, enhanced oxidative capacity is one of the most regarded. Indeed, a high SM mitochondrial density has been relentlessly highlighted as a univocal feature of SM-IS (Gouspillou et al., 2014; Roden and Shulman, 2019; Houzelle et al., 2020). Conversely, mitochondrial dysfunction has been related to loss of SM mass (Gouspillou et al., 2010) Furthermore, peripheral OXPHOS capacity is positively associated with an efficient glucose metabolism (Rimbert et al., 2004). In brief, elevated peripheral oxidative capacity is thought to be one of the strongest predictors of whole-body-IS in virtue of a higher total substrate utilization (Bruce et al., 2003). A recent study of Zampino et al. (2020) highlighted the influence of SM OXPHOS activity on resting metabolic rate, independently of total FFM, which suggests that intrinsic SM OXPHOS capacity is a stronger driver of energy expenditure than SM quantity (Zampino et al., 2020). Nonetheless, whether exercise-induced SM hypertrophy leads to significant mitochondrial adaptations with regards to improved IS remains unclear. A comprehensive study of St-Jean-Pelletier et al. (2017) has found a significant association between SM mitochondrial content and thigh lean mass and CSA, as well as lower body strength, in a pooled sample of young and older/sedentary or active men. Interestingly, they also found to significant relationship between mitochondrial and lipid content (St-Jean-Pelletier et al., 2017). Sparks et al. (2013) investigated the influence of either AT, RT or their adjunction (ATRT) matched for time in older individuals with T2D and found that ATRT has the most impact on mitochondrial content and substrate oxidation (Sparks et al., 2013). Interestingly, participants in the ATRT group displayed no significant changes in SM mass. This observation echoes Olver and Laughlin’s (2016) suggestion that with regards to exercise adaptations related to SM-IS, the combination of the two exercise modalities is more efficient than one of the two in isolation (Olver and Laughlin, 2016). According to the authors, this could be explained by a broader recruitment of the musculoskeletal system and potentially, a greater total exercise workload. Recently, Parry et al. (2020) reviewed the equivocal character of SM mitochondrial adaptations in response to RT regimens. In short, the authors proposed a novel theory, the “mitochondrial dilution” model, wherein SM hypertrophy without an equivalent rate of concurrent mitochondrial biogenesis leads to a decrease in relative mitochondrial content and ultimately, lowered or unchanged total oxidative capacity and mitochondrial respiration (Parry et al., 2020). Although the contribution of SM mitochondrial dilution on IS needs further investigations, one could hypothesize that since lower mitochondrial activity is a well-known feature of IR (Petersen et al., 2004), such a dilution could lead to a decrease in relative SM IS (Petersen et al., 2004). The authors also recommend examining how high-repetition/low-load or “aerobic-like” training, versus high-load/low-repetition or “hypertrophy-driven” training modulates markers of mitochondrial biogenesis. In line with those speculations, a study from Burd et al. (2010) has demonstrated that acute low-load, high volume RT, in terms of absolute number of repetitions has a greater impact on mitochondrial protein synthesis, compared to high-load – low volume RT (Burd et al., 2010).

A reduced turnover rate of intramuscular triglycerides (IMTG) is also a hallmark of SM-IR (Petersen et al., 2004; Roden and Shulman, 2019) and is thought to be the consequence of a low oxidative capacity (Consitt et al., 2009). Conversely, an elevated IMTG turnover rate is associated with greater SM-IS, irrespective of the magnitude of IMTG pools (Goodpaster et al., 2001). Although the relevance of both aerobic and resistance exercises is no longer to be proven regarding enhanced IMTG metabolism, it remains unknown whether quantitative changes in SM play a role in those molecular adaptations. However, it is generally accepted that hypertrophied SM contains proportionally more type IIA and IIB than type I fibers (Karp, 2001). Nonetheless, these types of muscle fibers are thought to display a lower oxidative capacity (Henriksson and Reitman, 1976). A study of Shepherd et al. (2014) provided insightful observation to this issue by revealing that RT has the ability to increase IMTG breakdown in type II fibers (Shepherd et al., 2014). In short, their results demonstrated an increase in lipid droplet-associated proteins 2 and 5 (PLIN2 and PLIN5) covering lipid droplets, as well as an increase in intramuscular triglyceride utilization during a 1-h moderate intensity cycling session after a 6-week RT protocol in lean, healthy young men. Furthermore, there was an increase in the expression of cytochrome oxidase (COX; a marker of SM oxidative capacity) in both type I and II fibers. There was also a slight increase in total SM mass. But most importantly, all adaptations occurred in both type I and type II muscle fibers. In sum, the current body of evidence suggests that even if the importance of AT and RT in glucose metabolism is no longer to be proven regarding IMTG metabolism, it has yet to be confirmed whether quantitative changes in SM mass further drives those adaptations in other populations, such as individuals with metabolic impairments.

### A Perspective Approach on the Optimal Strategy

Body weight management and exercise are amongst the most promising strategies to improve and maintain overall metabolic health (Tuomilehto et al., 2001). However, it is still unclear if the current focus on SM hypertrophy, or the prevention of SM losses during weight loss trials is appropriate amongst all populations. In contrast, studies aiming at increasing muscle quality, regardless of quantity, are currently scarce (Barbat-Artigas et al., 2013). This can be achieved with low-load-high repetition RT, which is an overlooked exercise modality in metabolically impaired populations. Further studies that will seek to determine the real contribution of quantitative changes of SM on glucose homeostasis in populations who would benefit from an optimal exercise prescription for this purpose, such as prediabetic, T2D or metabolically impaired individuals, are highly warranted.

## Conclusion

Regular exercise, be it AT or RT, has repetitively been associated with improved IS through various mechanisms. However, the mechanisms underlying a relationship between SM hypertrophy and whole-body glucose homeostasis have not been demonstrated without doubt. A fundamental mechanism that could contribute to IS improvements is greater SM capillarity (i.e., greater perfusion surface) and its vasodilator response. The associated increase in blood-flow and diffusion surface for O_2_ support improved SM OXPHOS capacity and intramuscular lipid turnover. We argue that RT protocols both involving a high O_2_ demand and aiming to improve muscle function have the highest potential to induce SM adaptations in favor to an improved IS. With this in mind, SM hypertrophy ability to equivocally improve whole-body glucose homeostasis might be re-evaluated in specific populations. Moreover, in virtue of better known intersex- and age- related physiological differences in response to acute and chronic exercise (Lundsgaard and Kiens, 2014; Hughes et al., 2015; Snijders et al., 2017; Ansdell et al., 2020), specific recommendations are warranted. Finally, while RT is a highly relevant and proven strategy to prevent T2D (Bird and Hawley, 2017; Pesta et al., 2017), future investigation should seek to determine which modality of RT would maximize such benefits and verify if RT, through improvements of SM quality, can substantially increase IS independently of SM mass changes.

## Author Contributions

ID and MB had the idea of the article and supervised JP in the writing. JP performed the literature search, article analysis and wrote the whole manuscript and tables. J-CL critically revised the manuscript and tables. All authors contributed to the article and approved the submitted version.

## Conflict of Interest

The authors declare that the research was conducted in the absence of any commercial or financial relationships that could be construed as a potential conflict of interest.
